# “Let’s Chat”: process evaluation of an intergenerational group chat intervention to increase cancer prevention screening among Vietnamese American families

**DOI:** 10.1093/tbm/ibaa120

**Published:** 2021-04-07

**Authors:** Huong T. Duong, Suellen Hopfer

**Affiliations:** Department of Health, Society, and Behavior, University of California, Irvine, CA

**Keywords:** Cancer screening, Colorectal cancer, Cervical cancer, HPV vaccination, Family communication, Group chat interventions

## Abstract

Vietnamese Americans have a higher rate of cervical and colorectal cancer (CRC) compared to other ethnicities. Increasing CRC screening, Pap testing, and HPV vaccination is critical to preventing disproportionate cancer burden among Vietnamese families. To describe the successes and challenges of implementing a novel intergenerational family group chat intervention that encourages CRC screening, Pap testing, and HPV vaccination. Young adult Family Health Advocates (FHAs) were trained to facilitate online family group chat conversations to encourage cancer screenings. Ten families participated in a 4-week intervention. Data collection included screenshot data of family group chat conversations, family member surveys, and post-intervention FHA interviews. Intervention implementation successes included (a) cultural and language brokering, (b) active co-facilitation by family members to follow up on cancer screenings, (c) high levels of family group chat engagement, (d) high acceptability of intervention among families, and (e) accessibility of intervention curriculum. FHA challenges to implement the intervention included (a) sustaining cancer prevention conversations, (b) comfort with navigating family conversations around cancer screening, (c) relevance for all family members, and (d) missed opportunities for correcting misinformation. Researcher challenges included family recruitment and retention. The intervention made cancer-screening messages more accessible and was well accepted by Vietnamese families. Scaling up the intervention will require (a) training FHAs to monitor family conversations and build confidence in sharing medical accurate messages, (b) segmenting group chats by age and gender, and (c) employing multiple family engagement strategies.

## INTRODUCTION

Cancer is the leading cause of death for Vietnamese Americans living in the United States [1]. Vietnamese women have the second highest cervical cancer incidence rate (9.5 per 100,000) among all Asian subgroups [2]. Colorectal cancer (CRC) also disproportionately affects Vietnamese Americans with incidence rates of 47.8 per 100,000 and 30.7 per 100,000 for men and women, respectively [1, 2]. Late screening contributes to these alarmingly high cancer rates among Vietnamese families, but the high cancer rates could be prevented through early prevention (e.g., Pap testing, HPV vaccination, and CRC screening). We designed *Let’s Chat*, an intervention aimed at reaching a hard-to-reach population using social media family group chats to deliver personalized cancer screening messages delivered by family health advocates (FHAs).

Intergenerational family communication and interpersonal communication have played important roles for increasing cancer screenings among Asian American families [3–5]. For example, prior studies indicate that having younger family members involved in communication and encouragement of recommended cancer screenings increased CRC and Hepatitis B screening among older family members [5, 6]. The recent adoption of social media messaging applications (e.g., SMS texting/iMessage, Facebook Messenger, Viber, WhatsApp, GroupMe, WeChat) into the intergenerational family context allows for communication to occur more frequently and easily [7, 8]. The composition of family group chats in Vietnamese families is often intergenerational, including grandparents, parents, aunts, uncles, siblings, and cousins as group members [9]. To the best of our knowledge, no intervention studies have incorporated intergenerational, mediated family conversations to increase family member cancer prevention behavior in the Vietnamese American population. Consequently, public health communication researchers have an opportunity to capitalize on this communication trend to share and reinforce important cancer prevention messages.

The theoretical foundation of *Let’s Chat* is grounded in (a) employing a lay health approach, (b) the Health Belief Model, and (c) cultural grounding [10–12]. Cancer screening interventions among the Vietnamese communities have successfully been applied using lay-health workers given their in-group trust with the community [11, 12]. This concept was adapted for the digital family communication environment to have young adult family members act as FHAs in their group chats and share cancer screening information, which can increase group trust in the advocated health message [13]. A lay health advocate model capitalizes on an already existing family group structure to have “insider” family members facilitate screening conversations and buy-in.

Constructs from the Health Belief Model guided the intervention by targeting perceived cancer susceptibility, severity, screening barriers and benefits relevant to Vietnamese American families, and cues to act on recommended screening [4]. Perceived susceptibility was enhanced by presenting cancer statistics relevant to the Vietnamese population. Self-efficacy messages were integrated by having family members share their personal screening experiences and share links to cancer screening resources. Cues to action were incorporated by FHAs verbally encouraging family members to make a cancer-screening appointment to talk with their doctor. The *Let’s Chat* intervention was also co-created by FHAs through a process involving cultural grounding.

Cultural grounding acknowledges the role of culture in health behaviors [10]. A cultural grounding approach to intervention design involves eliciting and co-creating cancer screening appeals that resonate with how the target audience ascribes meaning, in this case Vietnamese families’ own meanings, messages, and identities as it relates to cancer screenings [10]. The messages shared in the intervention were tailored using statistics and cultural cues relevant to the Vietnamese demographic. For example, screening messages were introduced at the onset of Tết, the Vietnamese new year celebrations in February to make connections with the importance of cancer screenings for staying healthy for the new year. In addition, FHAs were encouraged to tailor the delivery and sharing of cancer-screening messages on the group chat so that messages would resonate with their family culture.

The aim of this process evaluation was to examine the acceptability of the *Let’s Chat* intervention for Vietnamese families by (a) describing successes and challenges of implementing the intervention and (b) identifying practical approaches for implementing the intervention.

## METHODS

### Study procedures

Young adult Vietnamese Americans between the ages of 18–45 living in Orange County, California who self-reported having at least one family group chat were purposively recruited from a research university’s academic departments’ listserv via an emailed electronic advertisement. Young adults who met the eligibility criteria agreed to participate and in turn recruited family members who were active on their personal family group chats. Eligible family members had to have participated in the group chat within the last month as an indication of active group chat participation. Furthermore, each family group was required to have at least one family member who had never received recommended CRC screening including colonoscopy or FIT (by age 50), Pap testing (at age 21, every 3 years), or HPV vaccination (between age 18 and 45). After eligibility screening, FHAs created a new group chat with only family members who had consented to participate per IRB human subject protection recommendations.

### FHA training

Vietnamese young adults were trained as FHAs by attending a one-time, one-hour, in-person training session. FHAs were (a) briefed on the purpose of the intervention to share cancer-screening messages with family on their group chat, (b) asked about personal motivation for participating, (c) reviewed logistics of implementing the 4-week intervention (d) brainstormed ideas for engaging family members, and (e) reviewed how and what data to submit to the study researcher each week. A culturally tailored research website was created via weebly.com for (a) enrolling FHAs and (b) providing bilingual template cancer-screening messages prompts and information each week (e.g., infographics, videos, and websites to share with family groups). FHAs were not formally trained in how to conduct conversations, but were presented examples of conversation scenarios. They were highly encouraged to tailor the template messages according to their family’s needs to foster organic conversations. The training emphasized the FHA role was to educate, engage, and empower family members to follow up with recommended screenings. FHAs were instructed to submit de-identified screenshot captures of family conversations, a form documenting offline conversations about each week’s topics, and a reflection write-up of the week’s conversations including what went well and what did not in generating productive conversations. FHAs were compensated $100 for participation in the training, facilitating a 4-week intervention, and participation in an exit interview. Family members were offered a chance to participate in a raffle to win one of 10 $5 Starbucks gift cards after completing a post-intervention survey.

### Implementation procedures

The study was conducted between January and February 2018. During the first week, FHAs engaged family members by checking in with them about their overall health. Halfway through the first week, FHAs introduced CRC cancer screening by sharing recommending the colonoscopy as the gold standard for family members aged 50 and older. The second week involved discussions about alternative CRC screening such as the Fecal immunochemical test (FIT). The third week focused on introducing human papillomavirus (HPV) prevention by discussing vaccination recommended for young adults up to age 45. The fourth week focused on Pap testing for women aged 21 and older in addition to HPV vaccination for both men and women. Throughout the intervention, FHAs were reminded via weekly text messages to initiate each week’s cancer screening topic of conversation and submit screenshot data on weekly conversation. The researcher was available by text if FHAs had questions.

### Culturally targeted intervention cancer screening curriculum

Cancer incidence, screening messages, and supplemental information were adapted from publicly available tools from the Centers for Disease Control and Prevention, American Cancer Society, and the Asian American Network for Cancer Awareness Research and Training. Cancer statistics were reframed and tailored to be relevant for Vietnamese families. Examples of message tailoring for the template messages included addressing that feeling healthy does not replace screening [14]. Bilingual supplementary materials including culturally tailored infographics, videos, and website links were also available to disseminate based on the discretion of the FHA. Additionally, cancer prevention messages were introduced by employing Vietnamese cultural norms/etiquette to open the conversation each week by asking “Bác có khỏe không?” which directly translates into “Are you healthy?” or “How are you doing?” In addition, the intervention was launched during the Vietnamese Lunar New Year (Tết), which served as a cultural cue and conversation starter about being healthy in the new year. Table 1 shows sample messages offered as templates for FHAs to use to initiate conversation each week on the family group chat. FHAs were instructed during training to tailor the sharing of cancer messages to fit the context of family discussions.

### Data collection

Three types of process data were collected: Family conversation screenshots, family member online surveys, and post-intervention exit interviews with FHAs. The FHAs uploaded de-identified family conversation screenshots via a file upload form on the research study website. Family members were asked to participate in pre- and post-intervention online surveys sent by FHAs in the group chats. Surveys were available in English and Vietnamese. The survey included items asking family members’ intention to schedule recommended preventive cancer screening/vaccination, self-report screening follow-up, and self-efficacy in scheduling appointments and talking to their doctor. At the end of the survey, family members were asked to respond to two open-ended questions including: “What did you like most about participating in the family group chat discussions about cancer screening?” and “How could the discussions/educational content be improved?” These questions provided insight into family members’ perspectives of the implementation process. Post-intervention exit interviews were conducted with FHAs to understand their experiences initiating and sustaining family conversations about health, cancer, and preventive screening.

### Data analysis

Qualitative data from group chat conversations, family member surveys, and FHA interviews were content analyzed using NVivo 11 software [15]. Collecting and analyzing multiple sources of data was intentional to promote data triangulation [16]. Data from 10 families (41 participants) was analyzed. Ten family members did not return the post-intervention surveys and were therefore excluded from the final analysis. The majority of conversation data were already in English; however, 5 of the 10 families were bilingual. Of the five bilingual families, 21% of messages needed to be translated from Vietnamese into English for analysis. One entire family group chat did not return post-intervention surveys; however, data from their FHA’s exit interview were included. Group chat conversations were first read and re-read (data immersion) by two coders, followed by primary cycle line-by-line coding, tagging, and labeling content of family group conversations, reactions, responses, and questions [17]. This was followed by secondary hierarchical code organizing and identifying themes [17], grouped by intervention successes, FHA implementation challenges, and researcher challenges.

## RESULTS

### Participants

Ten families participated in the intervention (*n* = 41 participants). Family group chat size ranged from three to six members and included several generations (first, 1.5, and second generation) as well as several family members (e.g., parents, siblings, aunts, uncles, cousins). Among the 10 FHAs, 90% were female, 20 years was the mean age, all identified as second-generation immigrants, and all were currently enrolled in college. The majority (70%) reported intermediate Vietnamese proficiency while the remaining reported limited or advanced proficiency (1 reported limited; 2 reported advanced). Family group chat member age ranged from 18 to 61 with a mean age of 40. Out of the 41 family members, 31 participants completed pre- and post-intervention surveys. Eight (26%) identified as cousins, 3 (10%) as sisters, 2 (6%) as brothers, 7 (22%) as mothers, 4 (13%) as fathers, 4 (13%) as aunts, and 3 (10%) as uncles of the FHAs. All participants reported having health insurance, 29 (93.5%) had a primary care provider and 28 (90.3%) reported having a reliable form of transportation to get to their doctor’s office. Additional outcomes (under review) included family group chat engagement, family communication, intention to screen for CRC/vaccinate for HPV, and actual screening.

### Intervention successes

During the implementation process, we observed themes of engagement that naturally occurred and contributed to the success of the intervention. Themes of (a) cultural brokering, (b) co-facilitation, (c) family group chat engagement strategies, (d) acceptability, and (e) accessibility made the intervention engaging and well received by families.

#### Cultural and language brokering

Older family members typically preferred receiving cancer prevention material in Vietnamese. Young adult FHAs; however, struggled at times to convey medical information in Vietnamese. The FHAs attempted to accommodate by using Google Translate and attempted to write in both languages. However, there were often language barriers, in some group chats, family members stepped in to help translate medical information or facilitate by offering to make an appointment for the older family member. For example, one young adult cousin made an appointment for her father. This is an excerpt from their conversation:

Uncle D: I want to go [get screened]. L, come with me. I am scared.

Cousin L: Ok, dad. I will take you. Do you want to go talk with the doctor tomorrow?

Uncle D: What time?

Cousin L.: I will call and make appointment at 1 pm. Is that ok?

Uncle D: Thank you L and J (FHA).

#### Active co-facilitation by young adults to complete cancer screenings

There were several instances where young adult cousins or siblings stepped into the role of co-FHA by contributing information to the chat or providing their personal experiences. For example, one of the FHAs had a sister in the chat who was a medical resident and provided her expertise to co-facilitate the conversation. During the fourth week when discussing HPV vaccination, the conversation was very productive due to the FHA and the sister collaborating and co-facilitating as shown in this exemplar:

Mom: Hi FHA, even if saying HPV vaccine can prevent cancer. I didn’t have the vaccine when I was growing up. Too bad. You guys are lucky. I think you guys should have it so it can prevent cancer for your good, but I wonder if this vaccine has side effects in the future.

FHA: I don’t think vaccines have any side effects at all except you may get a little soreness in the arm, but it shouldn’t really have side effects. Because as far as I know, vaccines just contain the viral thing itself that’ll allow your body to fight against it and build immunity.

Sister C: Vaccines in most people don’t cause major reactions. Some can experience temporary fever or local pain from the shot. A few can have severe allergic reaction, which in that case is a reason to not get that particular vaccine in the future.

Co-facilitation by a family member with medical expertise also occurred in other chats on a smaller scale. For example, when family members needed clarification, other young adults weighed in to help clarify language barriers as shown in this example:

Uncle D: I don’t understand. What is screening?

Cousin V: Screening is a test for cancer.

FHA: Screening is “xét nghiệm cho ung thư.

#### Family group chat engagement

Family group chats that shared cancer screening information as question and answer format generated higher engagement compared to families where cancer-screening links were passively shared. In addition, some FHAs were more proactive than others when sharing health information with family members. Some FHAs facilitated family conversations by asking questions, sharing a link, and following up with a conversation about the topic content, while others merely sent the link for family members to read on their own. The latter strategy resulted in lower engagement in family conversations around cancer screening.

#### Family acceptability of intervention

FHAs reported enjoying the facilitation process of the intervention, emphasizing the opportunity to discuss cancer screening with their family members was unique. Many FHAs expressed they were surprised by how engaged their family members were on the group chat. For example, FHA Paula said: “*My family was very open, but no one has ever brought up the topic of cancer prevention before…Usually it’s a conversation between my aunts/uncles and their doctor, but they never talk to their kids about how to prevent cancer*.” In contrast, FHA Karen said, “*My family is open to talking about health so it didn’t feel hard. We’re just comfortable with each other in that way. We’ve talked about cancer before because we’ve had family members who have had cancer.*”

The majority of family members stated they appreciated the group conversations. One family member (28-year-old cousin of FHA Alina) wrote: “*I liked the fact that we were all discussing an important topic and that we learned things some of us didn’t know yet.”* Many family members expressed that they felt closer and more open to talking with their family members through the intervention. For example, FHA Eric’s 51-year-old father said, *“I liked seeing other family member’s perspectives [about cancer].”* In general, the majority of families expressed high acceptability of the intervention because it was interactive and involved their family members.

#### Accessibility of intervention curriculum

FHAs conveyed that the intervention materials were very accessible and that the group chat format was easy to manage given its online nature. They also mentioned that having the culturally tailored website for reference during the intervention was very helpful to them in the process of preparing for each week’s discussion, implementing the conversations, and submitting the screenshot data after each week’s conversation. FHAs also mentioned that the weekly text message reminders from the study researcher were helpful in reminding FHAs to initiate each week’s conversation starter. Since the researcher was also available by phone/text when they had questions or needed help with translation, some mentioned that it was helpful to have someone on call if they needed help.

Family members also expressed that the cancer screening information was accessible due to it being culturally tailored. For example, FHA Paula’s 58-year-old uncle wrote, “*I liked the Vietnamese websites, there was a lot of useful information.*” In addition to sharing information in Vietnamese, several different formats were used to share cancer screening information (e.g., infographics, PDFs, websites, videos, Q&A), which helped cater to different format preferences. For example, FHA Paula’s 52-year-old aunt wrote, *“I liked the pdf files more than the websites. I liked everything presented to me all at once instead of clicking through pages.*” The online group chat format contributed to the accessibility of the intervention. Several family members mentioned that the group chat was easier to process the cancer screening messages than talking face-to-face. For example, FHA Alina’s 50-year-old father wrote, “*I liked the open sharing forum [format]. [It provided] group support and was very helpful and informative.*” While there were many successes, there were also challenges experienced by family members and by the researchers in implementing the group chat intervention.

#### FHA implementation challenges

Challenges that FHAs experienced included (a) sustaining group chat conversations with their family over time and timing of delivering messages, (b) being comfortable in their FHA role to navigate family dynamics, (c) navigating cancer topics that were not always relevant to all group chat members, and (d) missed opportunities for correcting misinformation. Family members commented on a need for more translated material, larger family groups, more topics for men, and providing complementary in-person conversations. Challenges that FHAs expressed aligned with sentiments articulated by family members.

#### Sustaining conversations and timing of messages

Sustaining conversations during each week’s conversation was an anticipated challenge. Consequently, the researchers and FHAs co-created a list of potential probing questions to ask during training. Despite this effort, some FHAs mentioned that their messages were not being read by family members due to time conflicts. This led to difficulty in initiating and sustaining conversations among some families. For example, FHA Tammy said: “*I would send out messages later in the week because my family members tend to respond on the weekend.”* Even though timing seemed to be an issue for this particular group, she also mentioned that on the weekend, it was less challenging to sustain conversations because they were online at the same time. FHA Jennifer shared that for her, timing was not so much an issue, but her concern was that her family members began to ignore her messages after the first 2 weeks. She said*: “Naturally conversations die out in general. It’s not just the health topic, but they just won’t reply to me…I know my sister is in school so she didn’t respond and my mom just didn’t want to respond in the end. Maybe it was because my family chat group only consists of my sister, mom and myself and they didn’t want to talk all the time.*” Since the evolution of conversations could not be predicted, the FHAs had to be creative in sustaining conversations each week. Other times, the conversation naturally died out until the next topic was introduced the following week.

#### Family dynamics and comfort with facilitation

Family dynamics played an important role in whether family members were responsive. For example, FHA Lena said: *“The majority of my family group are on the older side so they were hesitant. They weren’t as proactive… I am personally shy when talking with my family. The least enjoyable part of being an FHA was asking personal questions [about their cancer screening status]. If I were to see them face-to-face, I would never do that.”* Lena felt like she could not connect with the older adults in the chat and echoed this sentiment several times. FHA comfort when facilitating conversations also affected whether the FHA probed or followed up with additional questions to keep the conversation going. FHA Alina said: *“I didn’t really know what to ask. Sometimes I feel like it wouldn’t have worked because of the age…”* From this interview with Alina, she was hesitant to probe more because she was uncomfortable with how her family would respond given the mix of age groups in her family chat.

#### Cancer topic relevance for all family members

Prior to the intervention, we recognized that some topics might not be applicable to all family members, but was nevertheless important for them to learn about. For example, even though men cannot receive Pap tests, it is important for them to learn about HPV and the Pap test. In addition, although colorectal screening is not routinely recommended for young adults, it was important for them to learn about ways to prevent CRC for their future health. Despite these intentions, implementing conversations with family about multiple cancer prevention topics was challenging. For example, FHA Alina said*: “The screening information would only be applicable for some age groups and family members but not others. Perhaps in future studies, there should be age restrictions for participation.”* Other participants echoed similar ideas about making sure the chat conversation was relevant for all participants to keep them engaged. FHA Clara said: *“Maybe it’s better to target older adults. For example, my brother didn’t really engage in conversation because talking about cancers like colorectal cancer—wasn’t really relevant to him. He already got the HPV vaccination so he didn’t really care to participate.”* While learning about screenings is warranted, it would be ideal to segment and target family members by age, gender, as well as screening/vaccination status.

#### Missed opportunities to clarify sharing of inaccurate health information

Throughout the group chat conversations, there were instances where FHAs had the opportunity to probe further or clarify misconceptions, but did not due to either lack of confidence or strong content knowledge. Since the intervention was implemented by FHAs, researchers did not have the opportunity to correct any potential misinformation. There were a few instances of misconceptions by family members that could have been addressed by the FHA during group chat discussions but were not. For example, when FHAs posted about HPV, older family members (age >45) said they had already gotten the vaccination, however, they may have confused HPV with HBV (Hepatitis B Virus vaccine), which also affects the Vietnamese American population at high rates. This was a missed opportunity by FHA to clarify.

Another misconception occurred when Pap screening was discussed. FHAs asked family members why they thought Vietnamese Americans have higher rates of cervical cancer. Two family group chats discussed the role of diet as a cause of cervical cancer. The point of the conversation was intended to guide family members toward discussing the importance of Pap screening to detect cervical changes early to be able to prevent cancer. FHAs had been instructed to focus on prevention behaviors (HPV vaccination/Pap test), but it was difficult for them to redirect the conversation at times. Instead, they affirmed diet as a cause of cervical cancer.

### Researcher challenges: recruitment and retention of families

From the researcher perspective, the most challenging aspect of intervention implementation was initially, recruitment of entire families, and subsequently, retention of all family members in staying actively engaged until the end of the 4-week intervention. Recruitment of entire families presented a major challenge with young adults initially expressing high interest to participate, but encountering problems recruiting their entire family. Once families were recruited, retention of family members’ active participation for the entire 4 weeks presented a challenge in some families. Ten participants (4 from one family) did not return the post-intervention surveys even though FHAs reminded them.

## DISCUSSION

Families increasingly use social media group chats to connect and coordinate family life [9, 18]. Using a family group chat intervention can facilitate the rapid dissemination of accessible and trusted cancer screening messages. Additionally, culturally tailoring messages (e.g., delivered during Tet new year), sending weekly text reminders, sharing cancer prevention material in Vietnamese and in different formats (e.g., videos, infographics, Q&A, website), and utilizing trusted, personal family networks collectively can make cancer screening messages more relevant to families. Group chats present the opportunity to reinforce the importance of cancer screening recommendations that their doctors may have recommended.

Successes observed from the *Let’s Chat* pilot study include high family engagement and acceptability to discuss the somewhat taboo topic of cancer and cancer prevention. Employing questions and answer formats, following up, having multiple family members contributing online simultaneously led to families feeling closer in some cases. Presenting Vietnamese demographic-specific cancer statistics, using trusted family members to deliver messages, and encouraging FHAs to tailor conversations to their family’s needs were strategies intended to address cancer as a taboo topic.

Challenges encountered by FHAs when implementing the intervention included (a) sustaining conversations across 4 weeks, (b) feeling comfortable introducing cancer screening with family, and (c) lacking comprehensive training to sustain conversation and provide accurate medical information. Challenges met by researchers included (a) recruitment of entire families and (b) retention of all family members completing post-intervention surveys. Lessons learned and suggestions are provided in Table 2.

### Lessons learned for implementing a family group chat intervention

#### Brokering and co-facilitation

Both cultural and language brokering were observed in family group chats, phenomena in immigrant populations where children of immigrants provide translation or explanations to their parents or older adults in their family [19]. Younger members co-facilitated on the group chats by translating or explaining medical information to older family members, which created a sense of closeness among family members. This relationship between language brokering and family closeness has also been observed in Latino and Chinese immigrant families [19, 20]. Since some of the older adults shared their screening status and asked questions about screening, their disclosure acts helped create a space for brokering. Cultural and language brokering functioned as natural engagement strategies that helped family conversations flourish in the group chat.

#### Facilitation check-ins and time tailoring

Checking in with facilitators often and giving constant feedback after each week may help FHAs improve their facilitation styles. Though a best practice for person-based interventions is participant autonomy, this can lead to varying styles and differing results [21]. Although some FHAs engaged their family in continual conversation, there were others who only sent written educational material and asked family members to read it on their own time. It became clear in the conversation screenshots that some FHAs were more able to engage families, while others were unsure how to sustain conversations due either to lack of training, time, or motivation. For other FHAs, timing proved to be a challenge; therefore, tailoring the timing of messages to the family’s preferences may also improve participation.

#### Comprehensive training for FHAs

Though each FHA participated in a 1-hour training, the main focus of the training was on (a) a brief review on cancer screening information, (b) motivations for participation, (c) logistics of submitting data, and (d) brainstorming ways to facilitate conversation. Offering a more comprehensive training program to better equip FHAs with stronger content knowledge, probing questions, and prepare FHA to effectively navigate family discussions may enhance engagement. Providing resources for the FHAs to refer to on their own may not be effective because there were instances where the FHA missed opportunities to clarify or correct misconceptions. Designing online modules to simulate delivering cancer screening information may also be helpful for FHAs. Lastly, if FHA commitment becomes a challenge when the intervention is scaled up, inviting a trained Vietnamese community health educator to join the group chat may complement the current FHA approach.

#### Segment group chat intervention by age and gender

Though the intergenerational composition of the group chats can generate important conversations between older and younger generations and insights, this same intergenerational exchange can present challenges. Limiting chats to similar age group may make the cancer screening messages more relevant for one particular group (e.g., CRC with those ages 45+, HPV with those ages 45 and under, cervical cancer with women ages 21–65). Segmenting group chats by age may also allow younger adults to discuss ideas openly without having to change their communication style based on who is in the group chat [22]. Chats can be divided up into similar age groups or similar family status (e.g., grandparents, parents/aunts and uncles, and cousins/siblings). Tailoring group chats to only include a similar age and/or family status in a group chat may facilitate higher levels of engagement and may be easier for FHAs to manage.

#### Partner with community organizations to recruit families

There were varying levels of participation by gender and age. Women were more easily recruited and were more engaged in conversations compared to men. Women, particularly mothers, have been found to be the “main agents of family communication,” while fathers tended to resist learning new technology [9], which could have been a reason for higher female participation. In addition, young adults had lower engagement during some weeks, possibly due to low motivation or time limitations since young adults are usually working and/or in school. We recommend for future studies to consider alternative recruitment and retention strategies. Researchers may consider directly contacting family members or including family members in addition to the FHA to increase recruitment. Actively engaging and involving extended family members with study team personnel (rather than indirectly through the FHA) early on may increase engagement and minimize attrition (i.e., returning post-intervention surveys). Participants may have neglected to return surveys because they were no longer engaged or participant study fatigue. Another possibility is that FHAs who were the direct point of contact may have not strongly encouraged the return of surveys after receiving their own compensation for facilitating the intervention. Consequently, using community-based participatory research (CBPR) methods to collaborate with community partners may help overcome recruitment challenges and minimize retention. The majority of Vietnamese families are immigrant families, who utilize community-based resources, and may already have established trust with community organizations [23]. Monetary or other incentives for family members may also facilitate greater levels of participation among family members.

#### Employ multiple channels and multimodal delivery strategies

Feedback from family members revealed that some would have preferred an additional in-person component. The purpose of the online, mediated context was to facilitate comfort disclosing private information, but also to save time. Since the group chats were intergenerational, some family members were less engaged perhaps due to lack of comfort in using the mediated context. Though family members do use the group chats to communicate regularly and coordinate family activities, each family member’s involvement varies. Future studies can incorporate modern engagement features, such as using video chat in addition to text formats, sharing real-time/personalized voice messages, or a mix of in-person and online communication to assist family members in feeling more comfortable with the intervention.

### Future directions

This process evaluation highlights the successes and challenges of implementing a family group chat interventions as well as suggests strategies for overcoming potential challenges. The purpose of this study was to incorporate cancer screening information into naturally occurring family group chats often used in today’s contemporary online settings. Results suggest that interjecting cancer screening discussions as a normative part of family discussions and sharing personalized cancer screening messages from a trusted family member can (a) increase family comfort and engagement discussing cancer screening discussion, and (b) increase action toward cancer screening behaviors.

Cancer prevention conversations rarely happen in the family setting, particularly among Vietnamese families where cancer discussions can be taboo and preventive care may not be normative. Consequently, this intervention shows promise to shift family conversation norms to include preventive, health-oriented discussions. A practical application would involve implementing this intervention as part of the curriculum in a health communication or other health education course as an experiential learning project for students in public health or other health science major [24]. Vietnamese family members may more readily participate if they perceive participation as part of a university curriculum and perceive the young adult family member as an expert in the topic.

Leveraging social media group chats to introduce and normalize preventive cancer screening conversations among Vietnamese American families reinforces the importance of following up with recommended cancer screenings. This type of intervention may also be suitable for other underserved Asian populations such as Chinese, Cambodian, Korean, and Japanese Americans who are also disproportionately impacted by preventable cancers. Careful consideration of the intervention process and cultural tailoring is warranted to ensure participant engagement. There is potential to scale-up the intervention; however, more work is needed to identify best practices for training FHAs, understanding ideal family group chat characteristics, designing messages, and employing different modes of online communication. More research is needed in this area to increase screening behavior and decrease the burden of HPV-related and CRC among Vietnamese American families.

## Figures and Tables

**Table 1 |:** Template messages for weekly conversations (available in English and Vietnamese)

Week 1A: checking in with family members	Hi everyone, thank you for agreeing to participate in this study with me. First off, I wanted to just see how everyone is doing? This study is to better understand how we can talk about cancer screening in families. So, I invite you to openly discuss these topics with me. Thank you!
Week 1B: Introduction to CRC risk, prevention, and screening	This week we will talk about colorectal cancer. Did you know Vietnamese people are at risk for colorectal cancer? Feeling healthy does not replace the need to get screened. If you have been screened, what was your experience like?
Week 2: Alternative screenings to colonoscopy	Happy New Year, everyone! If you are healthy at the beginning of the year, then you’ll be healthy the whole year. If you are 45+, it may be your time to get a CRC screening. Talk to your doctor about the many ways of getting screened. Are there any questions about this topic?
Week 3: HPV-related cancers & HPV vaccine	This week we will talk about HPV-related cancers prevention. The HPV vaccination prevents cervical/vaginal cancer in women, anal and throat cancer in both men and women, and penile cancer in men. It is recommended for both men and women until age 45. Talk to your doctor about getting vaccinated. What do you think might be reasons for not vaccinating?
Week 4: HPV vaccination/Pap testing for Vietnamese women	The rate of cervical cancer among Vietnamese American women is 40% higher than Whites. Cervical cancer can be prevented by getting an HPV vaccine and visiting your doctor for a Pap test. Why do you think the rate is higher among Vietnamese women?

**Table 2 |:** Suggestions for Let’s Chat intervention modifications in response to challenges

Suggestion	Modification examples
Facilitation check-ins	Researchers should initiate weekly check-ins during the intervention to ensure facilitation is progressing smoothly.
Time tailoring	Send messages on the weekend rather than weekday, depending on family preference.
Comprehensive training for FHAs	Extend training to more than one hour to cover more content and potential misconceptions.
Segment group chats by age and gender	Since cancer topics vary by age and gender, create group chats by age (e.g., young vs. older adults) and gender (males vs. female).
Partnerships as recruitment strategy	Community partnerships may help recruit whole family units and be more effective to have the family buy-in with the intervention
Employ multiple channels and multimodal delivery strategies	Sharing cancer screening messages using multiple channels and multiple modes of delivery may be more interactive (e.g., text, video conferencing, in-person, phone calls).

## Implications

### Practice:

*Let’s Chat* makes cancer screening information more accessible and reinforces the importance of cancer prevention through trusted family networks.

### Policy:

Policymakers should invest in family group chat interventions as a low-cost and scalable solution to increase cancer screening among minority populations.

### Research:

Future research should identify best practices for designing and implementing scalable family group chat interventions that increase cancer screenings.

## References

[1] Jin H, Pinheiro PS, Xu J, Amei A. Cancer incidence among Asian American populations in the United States, 2009–2011. Int J Cancer. 2016;138(9):2136–2145. 26661680 10.1002/ijc.29958 PMC5283572

[2] American Cancer Society. Special Section: Cancer in Asian Americans, Native Hawaiians, and Pacific Islanders (Cancer Facts & Figures 2016). American Cancer Society; 2016. Available at https://www.cancer.org/content/dam/cancer-org/research/cancer-facts-and-statistics/annual-cancer-facts-and-figures/2016/cancer-facts-and-figures-2016.pdf. Accessibility verified February 4, 2020.

[3] Lau DT, Machizawa S, Demonte W, Colorectal cancer knowledge, attitudes, screening, and intergenerational communication among Japanese American families: an exploratory, community-based participatory study. J Cross Cult Gerontol. 2013;28(1):89–101. 23263883 10.1007/s10823-012-9184-z PMC3580000

[4] Ma GX, Gao W, Fang CY, Health beliefs associated with cervical cancer screening among Vietnamese Americans. J Womens Health (Larchmt). 2013;22(3):276–288. 23428284 10.1089/jwh.2012.3587 PMC3601630

[5] Juon H-S, Rimal RN, Klassen A, Lee S. Social norm, family communication, and HBV screening among Asian Americans. J Health Commun. 2017;22(12):981–989. 29173103 10.1080/10810730.2017.1388454 PMC5809127

[6] Ersig AL, Williams JK, Hadley DW, Koehly LM. Communication, encouragement, and cancer screening in families with and without mutations for hereditary nonpolyposis colorectal cancer: a pilot study. Genet Med. 2009;11(10):728–734. 19707152 10.1097/GIM.0b013e3181b3f42d PMC2917812

[7] Kammerl R, Kramer M. The changing media environment and its impact on socialization processes in families. Stud Commun Sci. 2016;16(1):21–27.

[8] Pham B, Lim SS. Empowering interactions, sustaining ties: Vietnamese migrant students’ communication with left-behind families and friends. In Lim SS, ed. Mobile Communication and the Family. The Netherlands: Springer; 2016:109–126.

[9] Taipale S The big meaning of small messages. In Taipale S, ed. Intergenerational Connections in Digital Families. Cham, Switzerland: Springer International Publishing; 2019:87–101.

[10] Hecht ML, Krieger JLR. The principle of cultural grounding in school-based substance abuse prevention: the drug resistance strategies project. J Lang Soc Psychol. 2006;25(3):301–319.

[11] Nguyen BH, Stewart SL, Nguyen TT, Bui-Tong N, McPhee SJ. Effectiveness of lay health worker outreach in reducing disparities in colorectal cancer screening in Vietnamese Americans. Am J Public Health. 2015;105(10):2083–2089. 26270306 10.2105/AJPH.2015.302713 PMC4566531

[12] Taylor VM, Jackson JC, Yasui Y, Evaluation of a cervical cancer control intervention using lay health workers for Vietnamese American women. Am J Public Health. 2010;100(10):1924–1929. 20724673 10.2105/AJPH.2009.190348 PMC2936992

[13] Smith D Health care consumer’s use and trust of health information sources. J Commun Healthcare. 2011;4(3):200–210.

[14] American Cancer Society. Recommended Messages to Reach Asian Americans. 2017. Available at http://nccrt.org/wp-content/uploads/CRC-Communications-Asian-American-Companion-Guide-Final_B.pdf. Accessibility verified February 4, 2020.

[15] NVivo Pro 11 [software program]. Version 11. QSR International; 2016.

[16] O’Brien BC, Harris IB, Beckman TJ, Reed DA, Cook DA. Standards for reporting qualitative research: a synthesis of recommendations. Acad Med. 2014;89(9):1245–1251. 24979285 10.1097/ACM.0000000000000388

[17] Tracy SJ. Qualitative Research Methods: Collecting Evidence, Crafting Analysis, Communicating Impact. West Sussex, UK: Wiley-Blackwell; 2013.

[18] Lim SS. Mobile Communication and the Family—Asian Experiences in Technology Domestication. Dordrecht, Netherlands: Springer; 2016.

[19] Hua JM, Costigan CL. The familial context of adolescent language brokering within immigrant Chinese families in Canada. J Youth Adolesc. 2012;41(7):894–906. 21681583 10.1007/s10964-011-9682-2

[20] Guan S-SA, Nash A, Orellana MF. Cultural and social processes of language brokering among Arab, Asian, and Latin immigrants. J Multiling Multicult Dev. 2016;37(2):150–166.

[21] Yardley L, Morrison L, Bradbury K, Muller I. The person-based approach to intervention development: application to digital health-related behavior change interventions. J Med Internet Res. 2015;17(1):e30. 25639757 10.2196/jmir.4055 PMC4327440

[22] Noar SM. An audience-channel-message-evaluation (ACME) framework for health communication campaigns. Health Promot Pract. 2012;13(4):481–488. 21441207 10.1177/1524839910386901

[23] Katigbak C, Foley M, Robert L, Hutchinson MK. Experiences and lessons learned in using community-based participatory research to recruit Asian American immigrant research participants: CBPR to recruit Asian immigrants. J Nurs Scholars. 2016;48(2):210–218. 10.1111/jnu.12194 PMC5296612 26836035

[24] Holtslander LF, Racine L, Furniss S, Burles M, Turner H. Developing and piloting an online graduate nursing course focused on experiential learning of qualitative research methods. J Nurs Educ. 2012;51(6):345–348. 22533499 10.3928/01484834-20120427-03

